# Outcome after PSMA PET/CT based radiotherapy in patients with biochemical persistence or recurrence after radical prostatectomy

**DOI:** 10.1186/s13014-018-0983-4

**Published:** 2018-03-02

**Authors:** Nina-Sophie Schmidt-Hegemann, Wolfgang Peter Fendler, Harun Ilhan, Annika Herlemann, Alexander Buchner, Christian Stief, Chukwuka Eze, Paul Rogowski, Minglun Li, Peter Bartenstein, Ute Ganswindt, Claus Belka

**Affiliations:** 1 Department of Radiation Oncology, University Hospital, LMU Munich, Marchioninistr. 15, 81377 Munich, Germany; 2 Department of Nuclear Medicine, University Hospital, LMU Munich, Munich, Germany; 3 Department of Urology, University Hospital, LMU Munich, Munich, Germany; 4 Department of Therapeutic Radiology and Oncology, Innsbruck Medical University, Anichstr. 35, A-6020 Innsbruck, Austria; 5 German Cancer Consortium (DKTK), Munich, Germany

**Keywords:** Prostate cancer, PSMA PET/CT, Biochemical recurrence, Biochemical persistence, Radiotherapy

## Abstract

**Background:**

PSMA PET/CT visualises prostate cancer residual disease or recurrence at lower PSA levels compared to conventional imaging and results in a change of treatment in a remarkable high number of patients. Radiotherapy with dose escalation to the former prostate bed has been associated with improved biochemical recurrence-free survival. Thus, it can be hypothesised that PSMA PET/CT-based radiotherapy might improve the prognosis of these patients.

**Methods:**

One hundred twenty-nine patients underwent PSMA PET/CT due to biochemical persistence (52%) or recurrence (48%) after radical prostatectomy without evidence of distant metastases (February 2014–May 2017) and received PSMA PET/CT-based radiotherapy. Biochemical recurrence free survival (PSA ≤ 0.2 ng/ml) was defined as the study endpoint.

**Results:**

Patients with biochemical persistence were significantly more often high-risk patients with significantly shorter time interval before PSMA PET/CT than patients with biochemical recurrence. Patients with biochemical recurrence had significantly more often no evidence of disease or local recurrence only in PSMA PET/CT, whereas patients with biochemical persistence had significantly more often lymph node involvement. Seventy-three patients were started on antiandrogen therapy prior to radiotherapy due to macroscopic disease in PSMA PET/CT. Cumulatively, 70 (66–70.6) Gy was delivered to local macroscopic tumor, 66 (63–66) Gy to the prostate fossa, 61.6 (53.2–66) Gy to PET-positive lymph nodes and 50.4 (45–52.3) Gy to lymphatic pathways. Median PSA after radiotherapy was 0.07 ng/ml with 74% of patients having a PSA ≤ 0.1 ng/ml. After a median follow-up of 20 months, median PSA was 0.07 ng/ml with ongoing antiandrogen therapy in 30 patients. PET-positive patients without antiandrogen therapy at last follow-up (45 patients) had a median PSA of 0.05 ng/ml with 89% of all patients, 94% of patients with biochemical recurrence and 82% of patients with biochemical persistence having a PSA ≤ 0.2 ng/ml. Post-radiotherapy PSA ≤ 0.1 ng/ml and biochemical recurrence vs. persistence were significantly associated with a PSA ≤ 0.2 ng/ml at last follow-up.

**Conclusions:**

PSMA PET/CT-based radiotherapy is an effective local salvage treatment option with significant PSA response in patients with biochemical recurrence or persistence after radical prostatectomy leading to deferral of long-term ADT or systemic therapy.

## Background

^68^Ga-PSMA-HBED-CC (^68^Ga-PSMA) positron emission tomography/computed tomography (PET/CT) has emerged as the gold standard in staging prostate cancer patients with biochemical persistence or recurrence after radical prostatectomy compared to conventional imaging like computed tomography (CT) or magnetic resonance imaging (MRI) [1, 2] and choline PET/CT [3]. PSMA PET/CT results in a modification of treatment e.g. addition of antiandrogen therapy (ADT), enlargement of the irradiated volume or even omission of radiotherapy in the event of advanced, metastatic disease in a remarkable high number of patients (33.8–76%) with biochemical persistence or recurrence [4–10]. Unlike conventional imaging, PET with ^68^Ga-PSMA offers the unique possibility of visualising prostate cancer residual disease or recurrence at very low prostate-specific antigen (PSA) levels with 58.3% of PET-positive results found in a PSA range of 0.51–1.0 ng/ml [11–16]. Data stemming from retrospective dose escalation studies in patients with biochemical recurrence after radical prostatectomy confirmed that a higher radiation dose is significantly associated with a risk reduction in biochemical failure [17] and was not associated with a difference in acute grade 2 and 3 genitourinary or gastrointestinal toxicity in a single randomised study - SAKK 09/10 when irradiating the former prostate bed with 64 Gy vs. 70 Gy [18]. Since the advent of PSMA PET/CT, dose escalation to macroscopic tumor residual disease or recurrence is now more precisely possible and is potentially associated with a further improvement of biochemical recurrence-free survival. Currently, there is paucity of data regarding outcome after PSMA PET/CT-based surgical [19–21] or radiotherapeutic treatment [22–27] in patients with persistent or recurrent prostate cancer. Since February 2014, offering PSMA PET/CT to all patients with recurrent or persistent prostate cancer after radical prostatectomy at our department, we evaluated the outcome following PSMA PET/CT-based radiotherapy.

## Methods

### Study population

In February 2014, ^68^Ga-PSMA PET/CT prior to radiotherapy was introduced at our department as the standard diagnostic staging tool routinely utilised in prostate cancer patients. A total of 176 consecutive patients underwent PSMA PET/CT prior to radiotherapy. 129/176 (73%) patients received PSMA PET/CT due to biochemical persistence (52%) or recurrence (48%) after radical prostatectomy without evidence of distant metastases (Table 1). 47/176 (27%) patients were excluded from the analysis: In 20/47 patients PSMA PET/CT was performed in the primary setting and in 27/47 patients distant metastatic disease was diagnosed. All patients provided written informed consent to undergo ^68^Ga-PSMA PET/CT. This retrospective analysis was performed in compliance with the principles of the Declaration of Helsinki and its subsequent amendments [28] and was approved by the Ethics Committee of our Medical Faculty.

**Table 1:** Patients’ characteristics

	All patients	PSA relapse	PSA persistence
N	129	62	67
Median age	72 (47–83)	74 (50–83)	70 (47–83)
Tumor stage			
pT2	43 (33%)	35 (57%)	8 (12%)
pT3a	36 (28%)	17 (27%)	19 (28%)
pT3b	48 (37%)	8 (13%)	40 (60%)
pT4	2 (2%)	2 (3%)	–
Nodal stage			
pN0	79 (61%)	46 (74%)	33 (49%)
pN1	37 (29%)	8 (13%)	29 (43%)
pNx/cN0	13 (10%)	8 (13%)	5 (8%)
Positive surgical margins	60 (47%)	16 (26%)	44 (66%)
Gleason score			
6–7	70 (54%)	44 (71%)	26 (39%)
8–9	59 (46%)	18 (29%)	41 (61%)
Median PSA at RPE	10.85 (2.37–190)	8.55 (2.37–48)	18.05 (3.99–190)
Postoperative PSA	0.12 (< 0.03–40.13)	< 0.03	0.47 (0.08–40.13)
Months since RPE	33 (2–192)	61 (9–192)	8 (2–94)
Median PSA at PSMA PET/CT	0.62 (0.13–40.13)	0.44 (0.15–6.24)	0.90 (0.13–40.13)
Median PSA at PSMA PET/CT in PET positive pts	1.09 (0.14–40.13)	0.78 (0.27–6.24)	1.6 (0.14–40.13)
Median PSA at PSMA PET/CT in PET negative pts	0.3 (0.13–3.24)	0.3 (0.13–3.24)	0.34 (0.13–1.33)
PSMA PET/CT result			
Negative	51 (39%)	31 (50%)	20 (30%)
Fossa recurrence only	24 (19%)	16 (26%)	8 (12%)
Lymph node positive only	42 (33%)	12 (19%)	30 (45%)
Fossa and lymph node recurrence	12 (9%)	3 (5%)	9 (13%)

*Pts* patients, *PSA* prostate specific antigen, *RPE* radical prostatectomy, *PSMA PET/CT* prostate-specific membrane antigen positron emission tomography/computed tomography

### Treatment application and follow up

All patients received PSMA PET/CT as staging prior to radiotherapy. Treatment management following PSMA PET, e.g. initiation of ADT for PET-positive results, treatment of pelvic lymphatic pathways and simultaneous boost volumes to local recurrence or lymph node recurrence supplementary to the irradiation of former prostate was documented for each patient. Follow-up examination was first performed 3 months after irradiation and then every six to 12 months. Follow-up time was defined as the interval in months between radiotherapy and the last recorded PSA.

### PSMA ligand and PET/CT imaging

PSMA-HBED-CC was radiolabelled with ^68^Ga^3+^ from a ^68^Ge/^68^Ga generator system (GalliaPharm^®^, Eckert & Ziegler AG, Berlin, Germany) using an automated synthesis module (GRP, Scintomics GmbH, Munich, Germany) and pre-packed cassettes (ABX GmbH, Radeberg, Germany) as described previously for a different PSMA ligand by Weineisen et al. [29]. ^68^Ga-PSMA PET/CT images extending from the base of the skull to the mid-thigh were acquired. PET/CT scan was obtained with intravenous injection of iodine-containing contrast agent (Ultravist 300, Bayer Pharma AG, Berlin, Germany; or Imeron 300, Bracco, Konstanz; 2.5 mL/s; in portal venous phase) 60 min after almost simultaneous intravenous administration of 20 mg furosemide and ^68^Ga-PSMA (mean 205 megabecquerel (MBq)). Directly prior to the PET/CT scan, patients were implored to empty their bladders to minimise tracer accumulation.

### Image interpretation

PET/CT was interpreted by a consensus read by one nuclear medicine physician and one radiologist. Location of lesions was each determined by CT. PET-positive lesions were visually identified by ^68^Ga-PSMA uptake above background and not associated with the physiologic uptake. CT-positive nodes were defined by increased short axis diameter, loss of fatty hilum, or increased contrast enhancement. Local recurrence was identified by ^68^Ga-PSMA uptake and/or increased contrast enhancement in the prostate bed [30].

### Radiotherapy treatment

Planning CT was done in supine position. Patients were advised to have a full bladder and an empty rectum. All patients received radiotherapy with intensity modulated RT (IMRT) or volumetric arc therapy (VMAT) and image-guided RT (IGRT) techniques (2–5 times/week) using cone-beam CTs. Target delineation was performed according to the Radiation Therapy Oncology Group (RTOG) atlas for salvage prostate cancer. The clinical target volume (CTV) of the former prostate gland is defined superiorly as 5 mm above the inferior border of the vas deferens remnant, inferiorly as above the top of penile bulb, anteriorly by the pubic symphysis, posteriorly by the anterior rectum and laterally by the medial edge of the obturator internus muscle. Planning target volume (PTV) was derived by expanding the CTV by a 5–7 mm margin in all directions. Generally, an overall dose of 66 Gy with 2 Gy per fraction was applied to the prostate bed and 50.4 Gy with 1.8 Gy per fraction to the lymphatic pathways when PET-positive nodal involvement was detected. PET-positive pelvic lymph nodes were treated with a simultaneous-integrated boost (1.85–2.2 Gy per fraction). Likewise, a simultaneous integrated/sequential boost (2.0–2.14 Gy per fraction) was applied in case of local recurrence in PSMA PET/CT. The delineation of the gross tumor volume (GTV), i.e. local recurrence or suspicious lymph, nodes was based on 68Ga-PSMA uptake above background and increased contrast enhancement in the prostate bed and on increased short axis diameter, loss of fatty hilum, or increased contrast enhancement of the respective lymph nodes.

### Statistical analysis

For statistical analysis, SPSS Statistics 24 (IBM, New York, USA) was used. Time to event data was calculated using the Kaplan-Meier method. Differences between subgroups were compared using log rank test with a *p* value of < 0.05 considered statistically significant. Uni- and multivariate logistic regression analysis was used to identify predictors for treatment response.

## Results

### Patients’ characteristics and PSMA PET results

One hundred twenty-nine consecutive patients were included in this retrospective analysis on outcome after PSMA PET/CT-based radiotherapy for biochemical persistence (48%) or recurrence (52%) following radical prostatectomy. Evidence of metastatic disease was considered an exclusion criterion. Patients with biochemical persistence were significantly more often high-risk patients with higher overall pathologic tumor stage (60% pT3b; *p* <  0.05), pathologic pelvic lymph node involvement (43% vs. 13% pN1; *p* <  0.05), positive surgical margins (66% vs. 26%; *p* <  0.05) and higher Gleason Score 8–9 (61% vs. 29%%; *p* <  0.05). Biochemical relapse was predominantly associated with low- to intermediate-risk [pT2 (57%), pN0 (74%) and Gleason Score 6–7 (71%)]. All patients with biochemical recurrence had a non-detectable PSA postoperatively and a longer time interval until PSMA PET/CT vs. patients with biochemical persistence (61 vs. 8 months; *p* < 0.05). Overall, most patients (78/129; 60%) had PET-positive findings. Patients with biochemical recurrence were significantly more often PSMA PET negative (50% vs. 30%) or if PSMA PET positive, they showed more often evidence of local recurrence (26% vs. 12%; *p* < 0.05). On the other hand, patients with biochemical persistence had significantly more often PET-positive lymph nodes with (13% vs. 5%) or without evidence of macroscopic tumour in the prostate bed (45% vs. 19%). A negative PSMA PET scan was significantly associated with lower PSA levels: 76% of these patients had a PSA level ≤ 0.5 ng/ml compared to patients with a positive scan with 74% of these patients having PSA levels > 0.5 ng/ml. Baseline patients’ characteristics are shown in Table 1.

### Management of PET-positive lesions and patients’ outcome

**Fig. 1:**
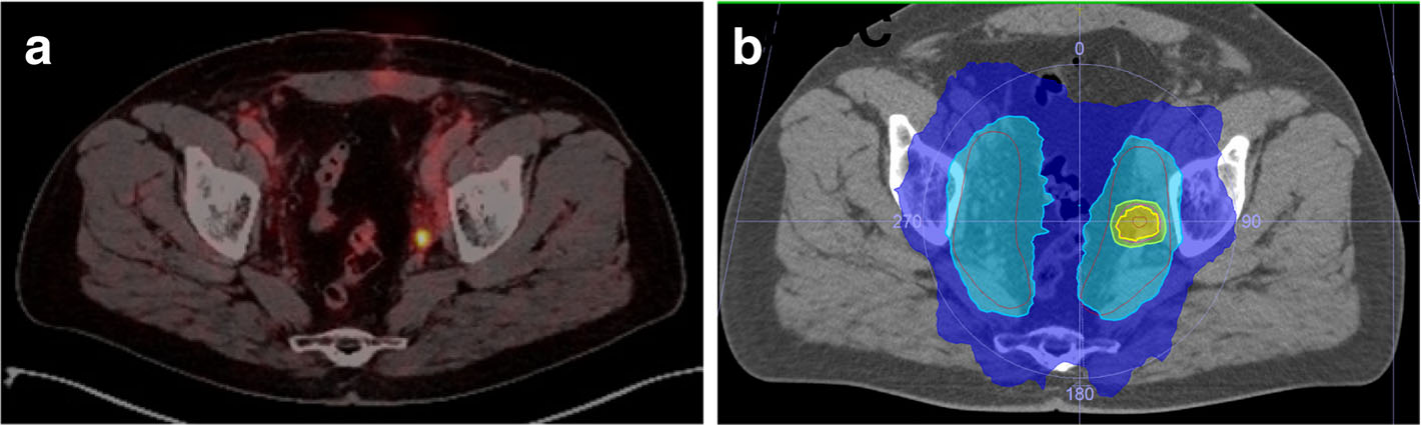
^68^Ga-PSMA PET/CT (**a**) and target volume with simultaneously integrated boost volume (**b**) to PET-positive lymph node

Androgen deprivation therapy (ADT) prior to radiotherapy was initiated in PET-positive findings (73/129). Five patients refused ADT. Fifty-nine percent (43/73) of these patients discontinued ADT after a median duration of 5 (2–25) months. All patients received VMAT- or IMRT-based radiotherapy to a median total dose of 66 (63–66) Gy to the prostate bed and 50.4 (45–52.3) Gy to the lymphatic pathways when PET-positive nodal involvement was detected with simultaneous integrated boost (SIB) to PET-positive lymph nodes (median dose 61.6 Gy; 53.2–66 Gy) (Fig. 1). In the case of local tumour recurrence/persistence, a SIB was delivered to a median total dose of 70 (66–70.6) Gy.

**Fig. 2:**
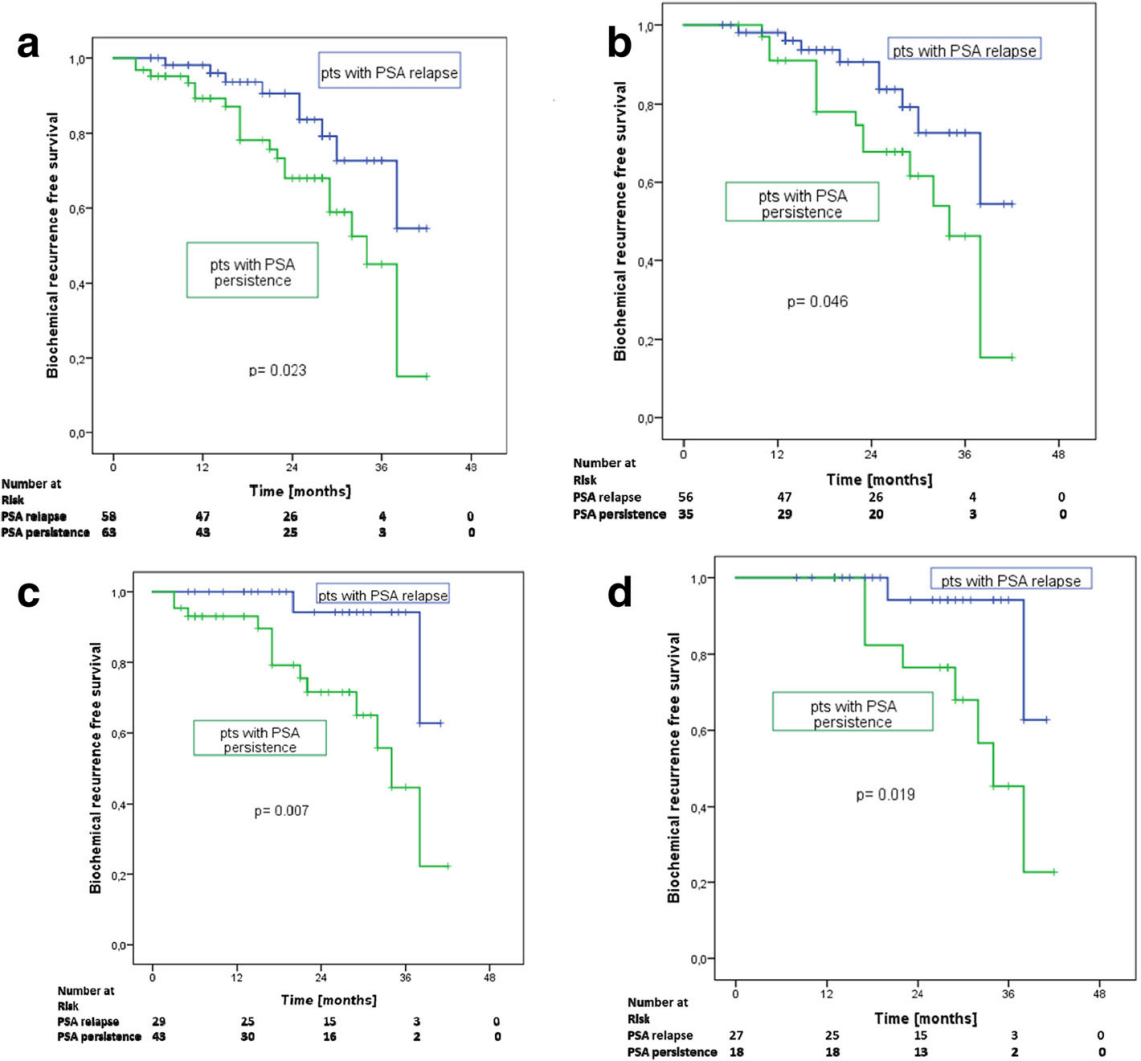
**a** Biochemical recurrence free survival (≤0.2 ng/ml) in all patients (PSA persistence vs. PSA recurrence) at last follow-up. **b** Biochemical recurrence free survival (≤0.2 ng/ml) in all patients without antiandrogen therapy (PSA persistence vs. PSA recurrence) at last follow-up. **c** Biochemical recurrence free survival (≤0.2 ng/ml) in all PET-positive patients (PSA persistence vs. PSA recurrence) at last follow-up. **d** Biochemical recurrence free survival (≤0.2 ng/ml) in all PET-positive patients without antiandrogen therapy (PSA persistence vs. PSA recurrence) at last follow-up

Median follow-up was 20 months (3–42). Median post-radiotherapy PSA was 0.07 ng/ml (< 0.03–13.71) with 74% of patients presenting with a PSA ≤ 0.1 ng/ml and 81% with a PSA ≤ 0.2 ng/ml. At last median follow-up, median PSA was 0.07 ng/ml (< 0.03–35) with 82% of all patients having a PSA ≤ 0.1 ng/ml and 84% a PSA ≤ 0.2 ng/ml (Fig. 2a). There were 30 (41%) patients with ongoing ADT after a median follow-up of 20 months with 79%/83% having a PSA ≤ 0.1/0.2 ng/ml, respectively. Patients without ongoing ADT (91 patients) had a median PSA of 0.07 ng/ml (< 0.03–35) with 83%/85% presenting with a PSA ≤ 0.1/0.2 ng/ml, respectively (Fig. 2b). PET-positive patients with discontinued or refused ADT at last follow-up (45 patients; 58%) had a median PSA of 0.05 ng/ml (< 0.03–35) with 89% of the entire cohort, 94% of the subgroup with biochemical recurrence and 82% with biochemical persistence having a PSA ≤ 0.2 ng/ml (*p* = 0.019; Fig. 2d). ADT at last follow-up was more often applied in the subgroup of patients with biochemical persistence than biochemical recurrence (93% vs. 7%; *p* < 0.05). Distant metastases at last follow-up were only detected in the subgroup with biochemical persistence (14/67; 21%). At the time of last analysis, all patients were alive. Treatment characteristics and PSA response are shown in Table 2 and Fig. 2.

**Table 2:** Treatment and response

	All patients	PSA relapse	PSA persistence
ADT			
ADT with stop before last follow up / duration (months)	43 / 5 (2–25)	22 / 4.5 (2–23)	21 / 5.5 (2–25)
Ongoing ADT at last follow up	30	3	27
No ADT	56	37	19
Median PSA before RT	0.62 (< 0.03–40.13)	0.44 (0.15–10.6)	0.90 (< 0.03–40.13)
RT			
Former Prostate	66 Gy (63–66)		
Lymphatic pathways	50.4 Gy (45–52.3)		
Local recurrence	70 Gy (66–70.6)		
PET pos. LN	61.6 Gy (53.2–66)		
RT technique			
VMAT/IMRT & IGRT	All pts.		
Median Follow-up (months)	20 (3–42)		
First PSA after RT	*n* = 121^a^	*n* = 58^b^	*n* = 63^b^
Median PSA	0.07 (< 0.03–13.71)	0.07 (< 0.03–0.56)	0.07 (< 0.03–13.71)
PSA ≤ 0.1 ng/ml	89 (74%)	47 (81%)	42 (67%)
PSA ≤ 0.2 ng/ml	98 (81%)	50 (86%)	48 (76%)
PSA at last median follow-up	*n* = 121^a^	*n* = 58^b^	*n* = 63^b^
Median PSA	0.07 (< 0.03–35)	0.07 (< 0.03–1)	0.07 (0.01–35)
PSA ≤ 0.1 ng/ml	82%	87%	77%
PSA ≤ 0.2 ng/ml	84%	91%	78%
PSA at last median follow-up with ADT	*n* = 30	*n* = 2	*n* = 28
Median PSA	0.06 (< 0.03–13.71)	< 0.03	0.07 (< 0.03–13.71)
PSA ≤ 0.1 ng/ml	79%	100%	79%
PSA ≤ 0.2 ng/ml	83%	100%	82%
PSA at last median follow-up without ADT	*n* = 91	*n* = 56	*n* = 35
Median PSA	0.07 (< 0.03–35)	0.07 (< 0.03–1)	0.08 (< 0.03–35)
PSA ≤ 0.1 ng/ml	83%	87%	78%
PSA ≤ 0.2 ng/ml	85%	91%	78%
PSA at last median follow-up in PET pos. pts	*n* = 72	*n* = 29	*n* = 43^b^
Median PSA	0.06 (< 0.03–35)	0.03 (< 0.03–0.46)	0.07 (< 0.03–35)
PSA ≤ 0.1 ng/ml	84%	94%	77%
PSA ≤ 0.2 ng/ml	86%	94%	79%
PSA at last median follow-up in PET pos. pts without ADT	*n* = 45	*n* = 27	*n* = 18
Median PSA	0.05 (< 0.03–35)	0.04 (< 0.03–0.46)	0.13 (< 0.03–35)
PSA ≤ 0.1 ng/ml	89%	94%	82%
PSA ≤ 0.2 ng/ml	89%	94%	82%
PSA at last follow-up in PET neg. pts	*n* = 49	*n* = 29	*n* = 20
Median PSA	0.07 (< 0.03–3.23)	0.07 (< 0.03–1)	0.08 (< 0.03–3.23)
PSA ≤ 0.1 ng/ml	78%	79%	77%
PSA ≤ 0.2 ng/ml	83%	87%	77%
Clinical Progress			
Distant Metastases			14

*PSA* prostate specific antigen, *ADT* androgen deprivation therapy, *LN* lymph nodes, *RT* radiation therapy, *VMAT* volumetric modulated arc therapy, *IMRT* intensity modulated radiotherapy, *IGRT* image guided radiotherapy

^a^PSA follow-up missing in eight patients

^b^PSA follow-up missing in four patients

### Factors predicting PSA response at last follow-up

An uni- and multivariate analysis was conducted to assess whether there was an association between tumour specific variables and PSMA PET/CT imaging results and a PSA ≤ 0.2 ng/ml at last follow-up. This was first studied in all 129 patients including all 30 patients on ADT at last follow-up (Table 3) and was repeated due to possible confounding bias in patients without ADT (99 patients; data not shown). Regardless of ADT usage at last follow-up, there was no association between a PSA ≤ 0.2 ng/ml at last follow-up and the following factors [ADT at time of irradiation, pre-RT PSA ≤/> 0.5 ng/ml, Gleason Score (6–7 vs. 8–9), pT- and pN-stage, surgical margins (positive vs. negative), PSMA PET result (negative or fossa recurrence only vs. PET-positive lymph nodes), PSMA PET result (positive vs. negative), overall dose ≤66.6Gy vs. >66Gy to the prostate fossa and overall dose ≤50.4Gy vs. > 50.4Gy to pelvic lymph nodes]. A post-radiotherapy PSA ≤ 0.1 ng/ml and biochemical recurrence vs. persistence were significantly associated with a PSA ≤ 0.2 ng/ml at last follow-up irrespective of ongoing ADT. On multivariate analysis, a significant association between post-radiotherapy PSA ≤ 0.1 ng/ml and PSA ≤ 0.2 ng/ml at last follow-up was observed irrespective of the study population.

**Table 3:** Association between treatment response (PSA ≤0.2 ng/ml) at last follow-up and possibly interacting variables (logistic regression analysis, all patients)

Association between treatment response and	*p*-Value^a^	*p*-Value^b^
ADT at time of RT	0.104	0.131
PRE-RT PSA ≤/> 0.5 ng/ml	0.226	0.188
Gleason Score (6–7 vs. 8–9)	0.129	0.791
pT stage	0.104	0.195
pN stage	0.341	0.908
Surgical margins	0.833	0.609
PSA after RT ≤ 0.1 ng/ml	0.008	0.007
PSMA PET result (Negative or fossa recurrence only vs. lymph node recurrence)	0.623	0.999
PSMA PET result (positive vs. negative)	0.263	0.693
Dose escalation prostate fossa ≤66.6Gy vs. >66Gy	0.324	0.122
Dose escalation pelvic lymph nodes ≤50.4Gy vs. >50.4Gy	0.371	0.999
RT indication (biochemical recurrence vs. biochemical persistence)	0.026	0.211

*PSA* prostate specific antigen, *ADT* androgen deprivation therapy, *PRE-RT PSA* PSA pre-radiotherapy, *RT* radiotherapy

^a^univariate and ^b^multivariate binary logistic regression analysis

*p < 0.05 statistically significant

## Discussion

PSMA PET/CT is currently the best available imaging technique to differentiate local relapse in the prostate bed, pelvic lymph node metastases or even metastatic disease in patients with biochemical persistence or recurrence [31]. Particularly, in patients considered for salvage radiation treatment, PSMA PET/CT has a high detection rate of prostate cancer lesions outside the prostatic fossa [15], corresponding to the number of men failing after salvage irradiation of the prostate fossa. Thus in 5%/19% of our patients treated due to biochemical failure, PSMA PET/CT revealed pelvic nodal involvement with/without local recurrence, respectively. As expected, patients with biochemical persistence being significantly more high-risk patients had a significantly higher rate of PET-positive pelvic lymph node metastases with/without local disease (13%/45%). This leads to a remarkable change in treatment e.g. the modification of radiation fields, dose escalation to macroscopic tumour lesions and initiation of ADT, as already extensively studied in current literature [4–9].

Furthermore, PSMA PET/CT is especially sensitive in identifying tumour recurrence at PSA levels well below 1.0 ng/ml [12], enabling radiotherapy initiation at PSA levels that are still considered curable [32]. Currently, no general recommendation for a PSA cut-off prior to postoperative staging with PSMA PET/CT exists, although some data suggest a PSA of 0.83 ng/ml as an optimal cut-off value [33]. In comparison, PET-positive findings were seen in our cohort at a slightly lower median PSA (0.78 ng/ml) in patients with biochemical recurrence vs. biochemical persistence (median PSA 1.6 ng/ml).

All patients with biochemical recurrence or persistence underwent PSMA PET/CT-based radiation treatment: absence of PET-positive disease resulted in irradiation of the prostate bed exclusively, whereas in the case of local recurrence a SIB was delivered. Additionally, presence of PET-positive pelvic lymph nodes resulted in irradiation of the pelvic basin with SIB to the involved nodes.

Extrapolated from data for pN+ patients treated with adjuvant radiotherapy and concurrent ADT [34, 35], ADT was recommended for 2 years with evidence of PET-positive lesions. Although, 73 patients were started on ADT, about two-thirds discontinued prematurely after a median time of 5 (2–25) months due to patients’ preferences.

Based on a median follow-up of 20 months and an overall number of 129 patients, our analysis shows the high impact of PSMA PET/CT on oncologic outcome and is in accordance with the currently limited number of analyses on outcome after PSMA PET/CT-based radiotherapy in persistent or recurrent prostate cancer with mostly shorter follow-up [22–26]: Our analysis shows that there is a high rate and long-lasting treatment response to irradiation based on pre-treatment PSMA PET/CT in patients with biochemical recurrence or persistence with 84% presenting with a PSA ≤ 0.2 ng/ml at a median follow-up of 20 months. When restricting the analysis to PET-positive patients without ADT at last follow-up (45 patients), the most challenging and interesting subgroup, 89% had a PSA ≤ 0.2 ng/ml. Splitting this subgroup further into patients with biochemical recurrence vs. persistence, the number of patients with PSA ≤ 0.2 ng/ml after a median follow-up of 20 months becomes even more divergent: 94% of patients with biochemical recurrence and evidence of PET-positive disease without ADT at last median follow up had a PSA level ≤ 0.1 ng/ml and ≤0.2 ng/ml vs. 82% of patients with biochemical persistence having a PSA ≤ 0.2 ng/ml (*p* = 0.019; Fig. 2d). This mainly reflects the fact that men with biochemical recurrence in our cohort had significantly more local relapses within the prostate fossa compared to men with biochemical persistence with significantly more pelvic nodal involvement in PSMA PET/CT. Emmett et al. [23] likewise reported on treatment outcome from PSMA PET/CT informed salvage radiation treatment in men with rising PSA following radical prostatectomy: based on a shorter median follow-up of 10.5 months, they also saw a high number of treatment response to radiotherapy (29/36 patients; 83%) when disease was confined to prostate fossa compared to patients with PET-positive nodal involvement (16/26 patients; 61%). Furthermore, the high number of 94% of PET-positive patients with biochemical recurrence having a PSA ≤ 0.1 ng/ml after 20 months in our cohort compares nicely to the analysis of Zschaeck et al. [22] on 20 recurrent high-risk prostate cancer patients with a median PSA of 0.15 ng/ml after a median follow-up of 29 months.

Achieving a post-radiotherapy PSA nadir ≤0.1 ng/ml and radiotherapy indication (biochemical recurrence vs. persistence) were the only factors associated with a PSA ≤ 0.2 ng/ml at last follow-up in our cohort. This is in accordance with the recent data by Bartkowiak et al. [36] demonstrating that men with undetectable post-radiotherapy PSA have lower rates of metastases and a better overall survival. Unlike the findings by Emmett et al. [23] that PSMA PET result (negative or fossa recurrence only vs. PET-positive lymph nodes vs. distant metastases) is predictive of treatment response to salvage radiotherapy, we did not see any correlation between PSMA PET result (negative or fossa recurrence only vs. PET-positive lymph nodes) and biochemical recurrence-free survival. This is most likely to due to the fact, that we restricted our analysis to non-metastasised patients in contrast to the study by Emmett et al. which included patients with metastatic disease. Furthermore, contrary to data from literature showing a strong association between pre-RT PSA and progression-free survival [32, 36], there was no such association between pre-RT PSA ≤/> 0.5 ng/ml and PSA ≤0.2 ng/ml at last follow-up in our cohort.

Our study has several limitations mainly as it is a retrospective study. Moreover, there was a low number of patients with biochemical recurrence that limits the statistical power of a multivariate analysis. Thus, a validation of our results within a larger cohort would be preferable.

Currently, PSMA PET/CT is the best diagnostic tool available for patients with rising PSA post-radical prostatectomy. Yet, it may still underestimate the true extent of disease in particular for the detection of small volume lymph nodes below 4 mm due to the inherent physical limitations of PET imaging [1, 37] as well as for lesions close to the prostate fossa overshadowed by the SUV and radioactivity concentration within the bladder [38, 39]. The implementation of novel 18F–labeled PSMA tracers may overcome this issue because of its low clearance via the urinary tract. Consequently, one third of our PET-negative patients, all treated with irradiation to the prostate fossa, failed biochemically at last follow-up. Since all data available consistently document that PSA control is significantly better when radiotherapy is commenced as early as possible [32, 40, 41], it is nevertheless not justifiable to wait until PSA is in an optimal range or surpasses a cut-off for diagnostic assessment.

## Conclusions

Currently, PSMA PET/CT is the best available imaging technique in patients with persistent or rising PSA after radical prostatectomy and detects a high number of lesions not confined to the prostate fossa. PSMA PET/CT enables tailoring of radiation treatment with adaptation of irradiated volumes. After a median follow-up of 20 months, nearly 90% of men with PET-positive lesions without any ongoing antiandrogen therapy at last follow-up did not show any evidence of biochemical recurrence after PSMA PET/CT-based radiotherapy leading to a potential deferral of ADT or systemic therapy.

## Abbreviations

^68^Ga-PSMA: ^68^Gallium-prostate-specific membrane antigen
ADT: Androgen deprivation therapy
CT: Computed tomography
CTV: Clinical target volume
GS: Gleason score
GTV: Gross tumor volume
Gy: Gray
IGRT: Image guided radiotherapy
IMRT: Intensity modulated radiotherapy
MBq: Megabecquerel
MRI: Magnetic resonance imaging
PCa: Prostate cancer
PET: Positron emission tomography
PET/CT: Positron emission tomography/computed tomography
PSA: Prostate specific antigen
pts.: Patients
PTV: Planning target volume
RT: Radiotherapy
SIB: Simultaneous integrated boost
VMAT: Volumetric modulated arc therapy
